# Gratitude and Subjective Well-Being among Koreans

**DOI:** 10.3390/ijerph17228467

**Published:** 2020-11-16

**Authors:** Jieun Yoo

**Affiliations:** Department of Theology, Anyang University, Anyang 13900, Korea; msje9295@anyang.ac.kr

**Keywords:** gratitude, subjective well-being, Korean adults

## Abstract

This study examined the relationship between gratitude and subjective well-being (life satisfaction, hope, and positive and negative affect) with individual demographic background (i.e., age, education level, perceived economic status, and religious affiliation) in a sample of 761 Korean adults participated from five universities in South Korea. Specifically, gratitude was still an essential element for the subjective well-being of Korean adults, although the meaning of gratitude under Confucian culture implies indebtedness and obligation. The relationship between gratitude and subjective well-being did not differ by gender. Implications for the subjective well-being of Koreans are discussed.

## 1. Gratitude and Subjective Well-Being among Koreans

The Korean phrase *sohwakhaeng*, which can be translated into “small but certain happiness,” has become increasingly popular in Korean society since 2018 [1]. This term was first introduced by a Japanese writer, Haruki Murakami, in his 1986 essay. In his writing, the philosophy of *sohwakhaeng* is based on a little happiness that an individual can get very easily in everyday life (e.g., a morning run, wake-up call from a loved one, favorite meal served warm, or simply watching a favorite movie). People do not need a significant amount of money for happiness. However, they can achieve happiness from little things. Recently, life has become exhausting for Koreans, especially young Koreans. Korea is facing an economic downturn. Over 20% of Koreans aged 25 to 29 years are unemployed, marking the highest employment rate among Organization for Economic Co-operation and Development (OECD) members [2]. The pressure to be successful in the face of greater economic inequality and the higher cost of living in Korea makes happiness seem unattainable [1]. As such, more Koreans are turning to *sohwakhaeng* or small but certain happiness for respite from the hardships of everyday life.

Considering Korea’s increasing interest in *sohwakhaeng*, gratitude is expected to become more important. People who are trying to achieve happiness from small things find or create satisfaction and gratitude in their routine lives. In recent years, a great deal of literature has suggested that gratitude is strongly related to all aspects of well-being [3,4,5,6,7,8]. Several studies have investigated the relationship between gratitude and well-being with specific samples, such as nursing students [9], clinical nurses [10], university students [11], and early adolescents [12] in Korea. The current study was conducted in response to these Korean social issues. It examined the association between gratitude and subjective well-being (life satisfaction, hope, and positive and negative affect) in a sample of Korean adults.

## 2. Gratitude and Subjective Well-Being in Korea

Gratitude is derived from the Latin root *gratia*, meaning graciousness, grace, or gratefulness [13]. Emmons and McCullough (2003) defined gratitude as “the perception of a positive personal outcome, not necessarily deserved or earned, that is due to the actions of another person” [3] (p. 377). People who perceive the help of others in their positive experiences express thankfulness and feel appreciative, and this generalized tendency of emotion is called gratitude disposition [14]. Thus, the meaning of gratitude is regarded as a personal trait or emotional state [15]. In many cultures and societies, gratitude has been highly valued in that it strengthens social relationships [16,17] and has even a moral affect [18].

Research has distinguished between the concepts of gratitude and indebtedness [19,20,21]. Some research understands the difference, with the norm of reciprocity to return an equal value to the donor [22], different motivations of benefactor [23], and perceptions of benefactor intention [20]. Gratitude is more related to a sense of indebtedness or obligation in interdependent societies like Asian countries than in independent societies like Western countries [24]. Japanese and Korean people tend to relate gratitude and indebtedness with feeling sorry and gratitude when being helped spontaneously [25,26,27].

Filial piety is a central value in Confucian culture, providing a view of the family system under which parents take care of their children and children have a debt or obligation toward their parents [28]. The family system under Confucian culture is based on a reciprocity between parents and children [29]. This sense of reciprocity is so strong that it is used to relate to others and extends and applied to the community. When one provides for a man, he is obligated to repay his or her kindness besides expressing words of love or appreciation. Thus, gratitude is displayed with indebtedness or obligation, providing for someone or performing acts and sharing appreciation emotions. Gratitude should not be considered separate from indebtedness or obligation duty under Confucian culture.

Studies have shown that people with high levels of gratitude tend to be happier and more satisfied in their lives [7,30,31,32]. However, few studies have considered the meaning of gratitude in Confucian culture [24,33]. Thus, this study aimed to identify whether gratitude, mixed with the concept of indebtedness or obligation based on reciprocity, is still an important factor in subjective well-being among Korean adults. Moreover, several studies have shown that women have an advantage over men with regard to benefiting from gratitude [34,35], as well as experiencing and expressing gratitude [34,36,37]. However, one study supported that gender differences were not found in gratitude and the relationship between gratitude and life satisfaction with Korean adolescents [38]. Therefore, this study also examined whether the relationship between gratitude and subjective well-being is experienced differently between genders.

## 3. Methods

### 3.1. Pariticipants and Procedure

The current study had a cross-sectional design. The procedure was confirmed with the Declaration of Helsinki [39]. For this study, 800 Korean adults who were enrolled in 2018 fall semester from five private universities in South Korea were recruited. They participated voluntarily and anonymously and were informed that they could refuse to participate in the survey at any time. In total, 761 valid and usable responses were obtained. Thus, the final sample size was 761. Of the 761 participants, 292 were male (38.4%) and 460 were female (60.4%), with 9 missing values for gender (1.2%). Participants’ age ranged from 17 to 73 years, with a mean age of 28.80 and a standard deviation of 13.609. Of the 761 participants, 68.1% identified themselves as Protestants, 4.7% as Catholic, 3.0% as Buddhists, and 24.2% as religiously unaffiliated. Participants’ education level varied as follows: 68 (8.9%) completed high school, 466 (61.2%) were currently enrolled in college, 174 (22.9%) had a bachelor’s degree, and 35 (4.6%) had a graduate degree. There were 18 (2.4%) missing values for education. Participants’ perceived economic status varied as follows: 20 (2.6%) were very low, 108 (14.2%) were rather low, 494 (64.9%) were middle, 118 (15.5%) were rather high, and 12 (1.6%) were very high. There were nine (1.2%) missing values for perceived economic status.

### 3.2. Measures

#### 3.2.1. Gratitude

Gratitude was measured with two gratitude scales. The Gratitude Questionnaire (GQ-6 [14] is a six-item self-report tool (e.g., “I have so much in life to be thankful for”) to measure the dispositional aspect of gratitude. Each item was answered on a seven-point response scale ranging from 1 (strongly disagree) to 7 (strongly agree). For the present sample, the internal consistency of the scale was acceptable (Cronbach’s α = 0.818). The Gratitude Adjective Checklist (GAC) [14] is a three-item self-report tool (e.g., three adjectives: grateful, thankful, and appreciative) to assess gratitude. Each item was answered on a five-point response scale ranging from 1 (not at all) to 5 (extremely). The students in the present study were asked to rate the amount they experienced each feeling “during the past few weeks.” Here, the internal consistency of the scale was acceptable (Cronbach’s α = 0.717).

#### 3.2.2. Subjective Well-Being

Subjective well-being was measured with general life satisfaction, hope, and recent emotional experience items. Satisfaction with Life Scale (SWLS [40], a five-item self-report tool (e.g., “I am satisfied with my life”), was used to measure individuals’ general life satisfaction. Each item was answered on a seven–point scale ranging from 1 (strongly disagree) to 7 (strongly agree). For the present sample, the internal consistency of the scale was acceptable (Cronbach’s α = 0.828). The Hope Scale [41], a 12-item self-report questionnaire, was used to measure hope in terms of a person’s perceived capacity to generate pathways to meet desired goals and motivation to undertake the routs toward the goals. Responses were scored on an eight–point scale ranging from 1 (strongly disagree) to 8 (strongly agree). For the current sample, the internal consistency of the scale was acceptable (Cronbach’s α = 0.827). The Positive and Negative Affect Scale (PANAS [42], a 20–item self-report tool, was used to measure the positive affect (PA) (e.g., joyful, delighted, and cheerful) and negative affect (NA) (e.g., scared, lonely, and sad). Each item was answered on a five–point scale ranging from 1 (not at all) to 5 (extremely). For the present sample, the internal consistency of scale was acceptable (Cronbach’s α = 0.833 for the PA and Cronbach’s α = 0.856 for the NA).

### 3.3. Analysis

In the initial descriptive, a *t*-test was performed. To test the hypotheses on the relationships between gratitude and subjective well-being, hierarchical regression analyses were conducted. The first model contained only individual background variables, while the second model introduced the gratitude variable. All analyses were performed using SPSS 18.0 (SPSS Inc., Chicago, IL, USA).

## 4. Results

### 4.1. Descriptive Statistics

The means, standard deviations, *t*-test results, and ranges of the variables in this study are presented in Table 1. The Korean female group had a significantly higher score in perceived economic status, the GAC scale in gratitude variables, and the PA scale in subjective well-being variables than the male group. The Korean male group had a higher score on the NA scale than the female group. Korean male and female groups reported the same levels of education, religious affiliation, GQ-6, SWLS, and Hope.

### 4.2. Regression Analyses

To test the relationships between individual background variables and subjective well-being, hierarchical regression analysis was conducted. First, age, education level, perceived economic status, and religious affiliation were entered as control variables. Second, the two scales of gratitude were entered. In general, gratitude significantly predicted four outcomes of subjective well-being for both groups. Table 2 and Table 3 provide the results of the hierarchical regressions for four outcomes (SWLS, Hope, PA, and NA) and show the βs for the variables at each step.

For the male group (see Table 2), age and education level decreased the likelihood of NA (β = −0.363, *p* < 0.001 and β = −0.139, *p* < 0.05, respectively), and perceived economic status increased the likelihood of SWLS (β = 0.138, *p* < 0.05). However, other individual background variables did not have significant effects on any outcomes in subjective well-being. After controlling for individual demographic background (i.e., age, education level, perceived economic status, and religious affiliation), GAC was a predictor of all four outcomes. Specifically, GQ-6 was a stronger predictor of SWLS and Hope. However, GAC was a stronger predictor of PANAS. That is, GQ-6 had positive effects on SWLS and Hope (β = 0.481 and 0.550, *p* < 0.001, respectively), and GAC had negative effects on PA and NA (β = −0.173, *p* < 0.01 and β = −0.224, *p* < 0.001, respectively). Overall, the final models displayed a substantial amount of variance in subjective well-being. That is, 8.8–36.8% of the variance in the outcome variables was explained by the individual background variables and gratitude variables.

For the female group (Table 3), age, perceived economic status, and religious affiliation (β = 0.112, *p* < 0.05; β = 0.0233, *p* < 0.001; and β = 0.112, *p* < 0.05, respectively) increased the likelihood of SWLS. Perceived economic status also increased the likelihood of Hope (β = 0.163, *p* < 0.05). Age decreased the likelihood of NA (β = −0.252, *p* < 0.001). After controlling for individual demographic background, GQ-6 was a stronger predictor of SWLS, Hope, and PA. However, GAC was a stronger predictor of PANAS, that is, GQ-6 had positive effects on SWLS, Hope, and PA (β = 0.0413, *p* < 0.001; β = 0.449, *p* < 0.001; and β = 0.182, *p* < 0.05, respectively); GAC had negative effects on NA (β = −0.239, *p* < 0.001). Overall, the final models displayed a substantial amount of variance in subjective well-being. That is, 3.7–35.5% of the variance in the outcome variables was explained by the individual background and gratitude variables.

## 5. Discussion

This study examined the relationship between gratitude and subjective well-being in a sample of Korean adults. One of the findings of this study was that the male group reported having a higher negative affect score. In contrast, the female group reported experiencing more gratitude adjectives (e.g., grateful, thankful, and appreciative) and a higher positive affect. This result is in line with previous research showing that the degree to which people experience and express emotions can differ between men and women [43]. This is also consistent with previous studies revealing that women experienced and expressed more gratitude [34,36,37], while men were expected to be less receptive to gratitude and evaluate their grateful feelings more critically [35].

The study results showed that male participants who were older and more educated reported significantly lower negative affect. However, those who had higher perceived economic status reported significantly higher life satisfaction. Female participants who were older, closer to religion, and had higher perceived economic status reported significantly higher life satisfaction. The higher the perceived economic status of female participants, the higher their levels of hope they experienced. The older they became, the lower the level of negative affect they had. The results showed that the common factor determining life satisfaction was perceived economic status for Korean men and women. Women who perceived better economic status had even more hope. Whereas Koreans have achieved rapid economic growth, which has led to a spectacular increase in per capita income, South Korea ranked one of the lowest levels of life satisfaction among OECD countries [44]. However, for the current research results, individuals’ perceived economic status rather than their absolute economic situation was likely to be associated with life satisfaction. This is concomitant with previous research showing that the degree of relative material affluence, not the objective material affluence, was the principal condition required for happiness in Koreans’ lives [45].

The results also showed that Korean women might have a stronger likelihood of engaging in religion than Korean men, and this made them more satisfied in their lives. Women are generally more affiliated with religion than men [46]. A wealth of literature has found that religious affiliation may influence subjective well-being and be beneficial to mental health outcomes [47,48,49,50,51,52,53,54,55]. For example, You, Yoo, and Koh [38] reported a positive relationship between different aspects of religious practices and mental health for Koreans. Religious attendance may expose believers to religious lessons that promote a positive aspect of their internal and external reality that can lead to happiness and psychological well-being [50,54]. In addition, religious attendance was associated with a higher level of happiness among Korean women and Protestants [56]. Thus, religious affiliation may be beneficial in improving Korean women’s satisfaction with life.

Another finding was that age lowered negative affect for both groups. Carstensen [57,58] explained a socioemotional selectivity theory that older individuals have higher levels of positive affect and lower levels of negative affect to maximize positive outcomes, such as social support or help, and to minimize negative outcomes, such as interpersonal conflict [59,60]. Lawton [61] suggested an optimization theory that older people tend to control emotions for the purpose of emotion optimization [62]. The key to emotion control is to avoid negative emotions and choose social situations that can provide enough static emotional and intellectual stimuli. In addition, Kim and Min’s study [63] showed that older Korean adults experienced less negative affect and more positive affect. Thus, this finding showed that Korean adults regulated their negative emotions for their emotional stability and well-being as they got older.

The results of this study revealed that gratitude was an important predictor of subjective well-being for both Korean male and female groups. As expected from previous literature [7,30,31,32], gratitude was associated with life satisfaction, hope, positive affect, and negative affect among Korean adults. The meaning of gratitude under Confucian culture was complicated by the concept of indebtedness and obligation [25,26,27], although the finding showed that gratitude was still an essential element for the subjective well-being of Koreans, especially those seeking *sohwakhaeng*. In addition, the relationship between gratitude and subjective well-being did not differ by gender. The only gender difference was that GAC was a negative predictor of positive affect in the group of Korean males. The GAC score for the male group was significantly lower than that of the female group, and Korean men might not have been familiar with expressing three adjectives (e.g., grateful, thankful, and appreciative). However, this result alone could not fully explain the finding related to GAC and the positive affect of Korean men. Thus, further studies are needed to investigate why GAC had a negative effect on positive affect for Korean men.

The findings of this study provide a need for the practical gratitude intervention for Korean adults. Korean clinicians and researchers might need to consider how gratitude potentially affects the operation of subjective well-being. Encouraging Korean adults to develop grateful feelings and express them in their daily lives through gratitude interventions could help them use more adaptive strategies to reduce their levels of stress and improve their subjective well-being. In Seligman and his colleagues’ research [64], the gratitude intervention caused significant positive changes in a short time. For female college students, the gratitude promotion program had a significant effect on the meaning of life and subjective sense of well-being [65]. Among a community sample of adults, the gratitude intervention confirmed the improvement of positive affect, subjective happiness, and life satisfaction, and the reduction of negative affect and depression symptoms [66]. Therefore, gratitude intervention is expected to be useful in promoting a sense of subjective well-being and reducing internalized disorders such as depression and anxiety. 

Despite these contributions, this study has several limitations. First, the participants exclusively consisted of college students and college graduates. Although the sample size was large, participants were recruited from five universities in South Korea. Thus, further research is required to include a more heterogeneous sample from demographic backgrounds. Second, the demographic background of the sample was limited in that the number of females was greater than the number of males. Future studies could collect more male data in order to be more representative of the whole Korean population. Finally, this finding did not consider that the two gratitude questionnaires, GQ-6 and GAC, were not designed for studying persons in Korea with non-Western cultural tradition, even though two instruments have been used widely. Therefore, future studies may use or develop other gratitude instruments for Koreans or people in non-Western cultures.

## 6. Conclusions

The present study is unique in examining how individuals’ demographic backgrounds affected subjective well-being in the relationship between gratitude and subjective well-being in a sample of Korean adults. In addition, this study found no gender differences in the relationship between gratitude and life satisfaction, hope, positive affect, and negative affect. The results of this study provide some practical implications for gratitude intervention that would be useful for promoting subjective well-being for Korean adults.

## Figures and Tables

**Table 1:** Mean values, standard deviations, *t*-test results, and range of study variables.

Variables	Male (*n* = 292)		Female (*n* = 460)		*t*-Test	*p*	Range
	Mean	SD	Mean	SD			
Individual backgrounds							
Age	28.85	12.85	28.51	13.89	0.336	0.737	17–73
Education	2.29	0.70	2.20	0.66	1.937	0.053	1–5
Perceived economic status	2.92	0.73	3.04	0.64	−2.255	0.024 *	1–4
Religion (Yes = 1)	0.77	0.42	0.76	0.43	−0.665	0.506	
Gratitude							
GQ-6	5.71	1.10	5.66	1.18	0.554	0.580	1–7
GAC	3.29	0.93	3.60	0.95	−4.296	0.000 ***	1–5
Subjective well-beings							
SWLS	4.96	1.05	4.84	1.12	1.342	0.180	1–7
Hope	5.76	1.27	5.61	1.21	1.586	0.113	1–8
PA	2.28	0.77	2.62	0.69	2.388	0.017 *	1–5
NA	2.56	0.66	2.42	0.65	2.857	0.004 **	1–5

Note. GQ-6, The Gratitude Questionnaire; GAC, The Gratitude Adjective Checklist; SWLS, Satisfaction with Life Scale; PA, Positive Affect; NA, Negative Affect. * *p* < 0.05, ** *p* < 0.01, *** *p* < 0.001.

**Table 2:** Hierarchical regression analyses: Effects of individual background and gratitude variables on subjective well-being (male group; *n* = 292).

Variables	Subjective Well-Being							
	SWLS		Hope		PA		NA	
	Model 1	Model 2	Model 1	Model 2	Model 1	Model 2	Model 1	Model 2
Individual backgrounds								
Age	−0.008	−0.078	−0.034	−0.090	0.079	0.099	−0.363 ***	−0.333 ***
Education	0.088	0.073	0.114	0.084	0.096	0.083	−0.139 *	−0.153 *
Economic status	0.138 *	0.097	0.071	0.012	0.017	0.004	0.043	0.033
Religion (Yes = 1)	0.125	0.026	0.070	−0.031	0.132 *	0.122	0.013	0.014
Gratitude								
GQ-6		0.481 ***		0.550 ***		0.146 *		0.111
GAC		0.227 ***		0.121 *		−0.173 **		−0.224 ***
F (*df*)	3.037 (4)	25.063 (5)	1.601 (4)	24.857 (5)	3.598 (4)	4.218 (5)	15.405 (4)	13.517 (5)
R^2^ (ΔR^2^)	0.045 (0.030)	0.368 (0.354)	0.023 (0.009)	0.356 (0.342)	0.052 (0.037)	0.088 (0.067)	0.189 (0.177)	0.236 (0.219)

* *p* < 0.05, ** *p* < 0.01, *** *p* < 0.001. β = Standardized coefficient. Model 1 contained individual background variables; Model 2 contained individual background and gratitude variables.

**Table 3:** Hierarchical regression analyses: Effects of individual background and gratitude variables on subjective well-being (female group; *n* = 460).

Variables	Subjective Well-Being							
	SWLS		Hope		PA		NA	
	Model 1	Model 2	Model 1	Model 2	Model 1	Model 2	Model 1	Model 2
Individual backgrounds								
Age	0.112 *	0.024	−0.027	−0.097 *	0.065	0.066	−0.252 ***	−0.203 ***
Education	−0.004	−0.048	0.058	0.006	−0.001	−0.007	−0.069	−0.049 *
Economic status	0.233 ***	0.111 **	0.163 *	0.058	0.035	0.023	−0.079	−0.026
Religion (Yes = 1)	0.112 *	0.056	0.098	0.034	0.049	0.019	0.044	0.033
Gratitude								
GQ–6		0.413 ***		0.449 ***		0.182 *		0.017
GAC		0.240 ***		0.173 ***		−0.072		−0.239 ***
F (*df*)	9.788 (4)	36.459 (5)	4.428 (4)	29.290 (5)	1.016 (4)	2.597 (5)	8.593 (4)	9.749 (5)
R^2^ (ΔR^2^)	0.089 (0.080)	0.355 (0.346)	0.041 (0.032)	0.298 (0.288)	0.010 (0.000)	0.037 (0.023)	0.078 (0.069)	0.126 (0.113)

* *p* < 0.05, ** *p* < 0.01, *** *p* < 0.001. β = Standardized coefficient. Model 1 contained individual background variables; Model 2 contained individual background and gratitude variables.
